# Interventions to Reduce Loneliness in Informal Caregivers of People With Dementia: A Systematic Review

**DOI:** 10.1111/jocn.70382

**Published:** 2026-06-02

**Authors:** Tianxue Hou, Yuqian Luo, Mu‐Hsing Ho, Xiaohang Liu, Yuelin Li, Chia‐Chin Lin

**Affiliations:** ^1^ School of Nursing, Li Ka Shing Faculty of Medicine The University of Hong Kong Hong Kong China; ^2^ School of Nursing The Hong Kong Polytechnic University Hong Kong China; ^3^ Kiang Wu Nursing College of Macau Macau China; ^4^ Alice Ho Miu Ling Nethersole Charity Foundation Professor in Nursing Hong Kong China

**Keywords:** dementia, informal caregiver, loneliness, older adults

## Abstract

**Aim:**

To summarize the current evidence on reducing loneliness among informal caregivers of people with dementia, such as family members or friends.

**Design:**

A systematic review.

**Methods:**

The methodological quality was evaluated using the revised Cochrane risk‐of‐bias tool for randomized controlled trials and the revised JBI critical appraisal checklist for quasi‐experimental studies. Data were extracted as predefined and synthesized narratively. The Template for Intervention Description and Replication checklist was used to report the intervention characteristics.

**Data Sources:**

Six electronic databases (MEDLINE via PubMed, EMBASE, Cochrane Library, PsycINFO, CINAHL Plus, and Web of Science Core Collection) were searched for studies published in peer‐reviewed English journals from the inception of each database until 28 January 2024.

**Results:**

Eight studies were included in this review, published between 2002 and 2023, with three being randomized controlled trials. All included interventions were psychosocial. Only one study reported significant improvements in loneliness. Five studies utilized remote and online interventions, such as social networking, psychotherapy, and online social support. Interventions varied in their impact on secondary outcomes, including stress, depressive symptoms, anxiety, and caregiver burden. Four studies demonstrated a positive effect on caregiver stress levels. One pilot trial reported a positive impact on depressive symptoms, and another study noted potential improvements in anxiety. One pilot study reported an average improvement in caregiver burden.

**Conclusion:**

While the evidence is insufficient for conclusive statements, this systematic review suggests potential benefits of interventions to reduce loneliness and improve mental health among these caregivers. It highlights the promise of remote interventions in addressing loneliness among dementia caregivers.

**Implications for the Profession and/or Patient Care:**

The findings suggest that tailored interventions, especially those delivered remotely, can enhance the support provided to caregivers, potentially improving their mental health and overall well‐being.

**Reporting Method:**

This systematic review adhered to the PRISMA statement.

**Patient or Public Contribution:**

No patient or public contribution.

## Abstract (highlights)

**Highlights:**

- What problem did the study address?
  ○ The study summarized the interventions to reduce loneliness experienced by informal caregivers of individuals with dementia, which can significantly impact their mental health and well‐being.
- What were the main findings?
  ○ The main findings indicate that while some interventions did not yield statistically significant results, some remote interventions showed promise in reducing loneliness and improving mental health outcomes, such as stress and caregiver burden.
- Where and on whom will the research have an impact?
  ○ The research will have a substantial impact on informal caregivers of people with dementia, particularly in nursing and clinical settings where caregivers often seek support. It emphasizes the need for tailored interventions that can be integrated into routine care practices.

**What Does This Paper Contribute to the Wider Global Clinical Community?:**

- The findings suggest that nurses and clinicians can play a crucial role in implementing and promoting remote interventions tailored to the specific needs of caregivers. By understanding the unique challenges faced by caregivers, healthcare professionals can provide more effective support and resources.
- By addressing caregiver loneliness, nurses and clinicians can help improve the overall mental health and well‐being of caregivers, which in turn can enhance the quality of care provided to individuals with dementia. This holistic approach can lead to better health outcomes for both caregivers and patients.
- Nurses and clinical professionals can explore innovative intervention strategies that can be adapted to meet the diverse needs of caregivers.

## 1 Introduction

The global burden of dementia is increasing rapidly (Imahori et al. 2022). As the population ages, the number of dementia cases is expected to reach 131 million by 2051, posing significant challenges for healthcare systems and society (Prince et al. 2015). The progression of dementia generally leads to irreversible cognitive decline (Huang et al. 2020). People in the middle to late stages of dementia often experience impaired judgement, disorientation, and difficulty with understanding and communicating (Alzheimer's's 2016). Due to the worsening condition, individuals with dementia become more dependent on their caregivers, who are predominantly informal caregivers—family members or friends providing unpaid care and support (Alzheimer's's 2016). In the United States, approximately 83% of the care provided to people with dementia comes from informal caregivers (Friedman et al. 2015). The demands of caregiving, coupled with insufficient time for personal needs, can detrimentally affect caregivers' health (Bott et al. 2017), leading to both impaired physical functioning (e.g., decreased immune function, physical illness) and psychological problems (e.g., depression and loneliness) (Kiely et al. 2008).

Loneliness is defined as a subjective or perceived deficiency in the quality or quantity of social relationships, and it can be one of the most painful individual experiences (Ernst and Cacioppo 1999). Among informal caregivers of individuals with dementia, loneliness is a prevalent concern, affecting more than two‐thirds of them (Victor et al. 2021). Furthermore, loneliness strongly predicts depressive symptoms, highlighting its significant impact on caregivers' mental health (Saadi et al. 2021). The increased responsibilities often limit caregivers' social interactions (Vasileiou et al. 2017), leading to social isolation and exacerbating mental health issues (Bastawrous 2013). Given the growing population of people with dementia and the overwhelming stress on informal caregivers, designing effective interventions to adequately support informal caregivers is crucial (Kovaleva et al. 2018).

Psychosocial interventions aimed at reducing loneliness can be classified into four main categories: enhancing social skills, providing social support, increasing opportunities for social interaction, and addressing maladaptive social cognitions (Hawkley and Cacioppo 2010). For instance, engaging coaching interventions have shown promise in addressing loneliness by enhancing social connections for informal caregivers of individuals with dementia (Van Orden et al. 2023). Conversely, another study highlighted the ineffectiveness of befriending and peer support schemes in reducing loneliness, calling for more targeted interventions (Charlesworth et al. 2008). Despite existing research, a significant gap remains in systematically identifying and evaluating effective interventions specifically tailored to reduce loneliness in this group. Clarifying intervention types, delivery methods, and their reported outcomes may help develop support strategies that are appropriate for informal caregivers of people with dementia, including those caring for individuals with different dementia diagnoses.

Therefore, a systematic review focusing on interventions to reduce loneliness in informal caregivers of people with dementia is essential not only to summarize current evidence but also to identify promising approaches that may help develop and refine future interventions. While the findings may be preliminary, they can contribute to shaping more targeted interventions for informal caregivers of people with different types of dementia.

### 1.1 Aim

To our knowledge, no systematic review has specifically focused on loneliness in informal caregivers of people with dementia. Understanding the effectiveness of interventions to reduce loneliness can inform further research and the development of supportive measures. This systematic review is the first study to summarize the current evidence on interventions targeting loneliness in these caregivers. We aimed to (1) identify and describe the current interventions aimed at reducing loneliness among informal caregivers of people with dementia, and (2) evaluate their effectiveness based on reported outcomes such as loneliness, stress, depression, and caregiver burden, using validated assessment tools to support future research in developing effective interventions for this population.

## 2 Methods

### 2.1 Design

This systematic review adheres to the Preferred Reporting Items for Systematic Reviews and Meta‐analyses (PRISMA) statement. The review protocol and records are accessible online through the Prospective Register of Systematic Reviews (PROSPERO: CRD42024500648).

### 2.2 Search Strategy

We conducted a comprehensive search across the following databases: MEDLINE via PubMed, EMBASE, Cochrane Library, PsycINFO, CINAHL Plus, and Web of Science Core Collection. The search covered the period from the inception of each database until 28 January 2024. We used search terms in three categories: (1) loneliness; (2) informal caregivers; (3) dementia. Supporting Information S1 provides an example of the search strategy used for PubMed.

**FIGURE 1:**
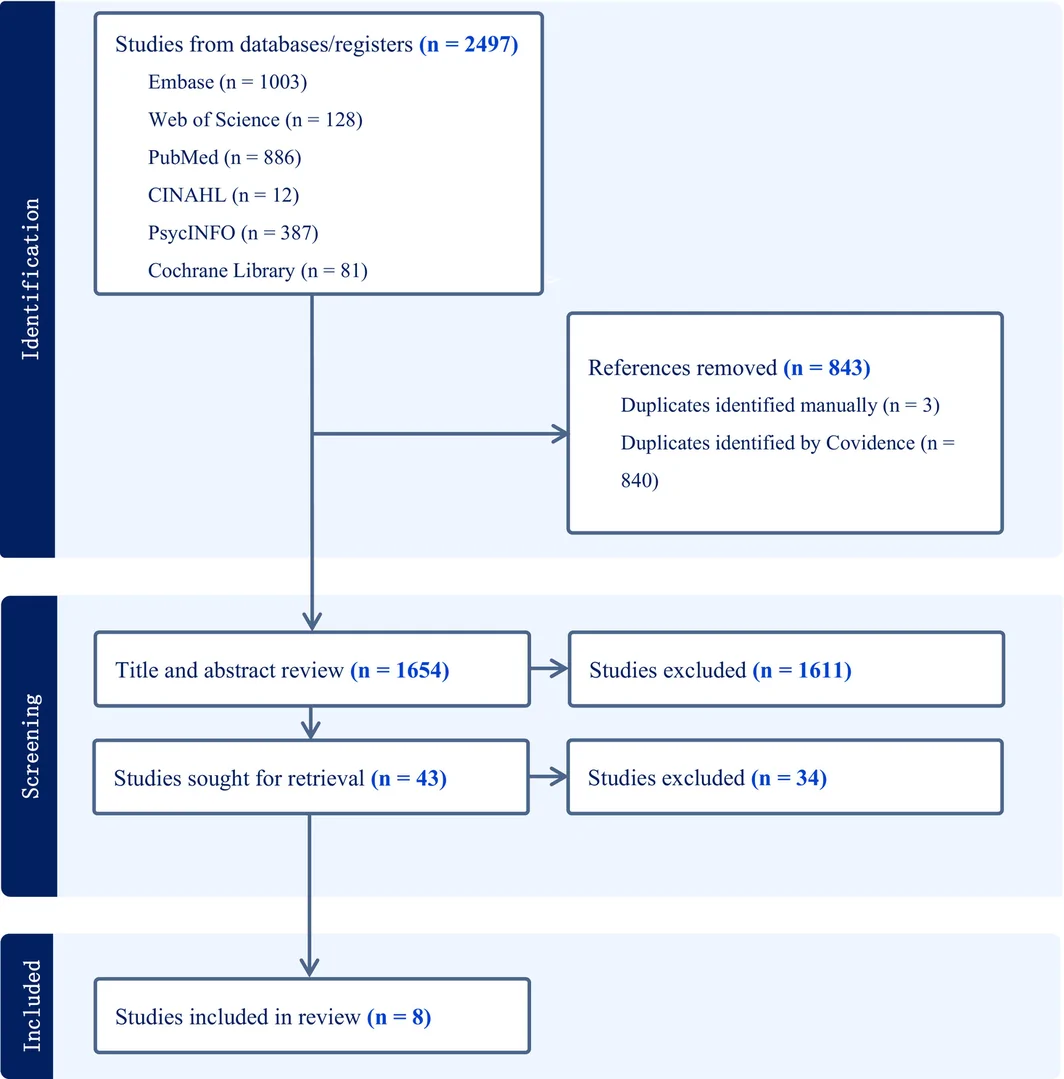
Flow diagram detailing the selection process of the included studies. [Colour figure can be viewed at wileyonlinelibrary.com]

The search results were imported into EndNote 21.0. Two reviewers (TH and YL) independently screened the titles and abstracts, excluding studies that did not meet the inclusion criteria. Full‐text reviews were conducted for potentially eligible articles. Any disagreements between the two reviewers were resolved by a third reviewer (MH). The screening outcomes are documented and presented in a PRISMA flow diagram (Figure 1).

### 2.3 Inclusion and Exclusion Criteria

Inclusion criteria were developed using the population, intervention, comparison, outcome, and study design (PICOS) format. Studies were included if they met the following criteria: (1) involved informal caregivers of people with dementia; (2) featured any non‐drug intervention aimed at reducing loneliness; (3) measured loneliness using any recognized measurement or clinical survey; (4) employed study designs such as randomized controlled trials (RCTs), non‐RCTs, pilot studies, quasi‐experimental studies, or pre‐post experimental studies; and (5) were published in peer‐reviewed journals in English. We did not include comparison as a criterion. Review articles, meta‐analyses, theses, and conference proceedings were excluded.

### 2.4 Data Extraction and Quality Assessment

One reviewer (TH) independently extracted study characteristics using a pre‐developed standardized electronic form, and another reviewer (YL) verified the accuracy. Extracted data included the author, year, country, caregiver age, study design, sample size, inclusion and exclusion criteria, duration, intervention details, and assessment tools. The Template for Intervention Description and Replication (TIDieR) checklist was used to guide the extraction of intervention characteristics, which included the rationale, what materials were used, what procedures, who provided the intervention, how, where, when and how much the intervention was delivered, any intervention tailoring and modifications, and how well the intervention was delivered (Hoffmann et al. 2014).

We assessed the methodological quality of all studies using the revised Cochrane risk‐of‐bias tool (RoB 2.0) for RCTs (Sterne et al. 2019), and the revised JBI critical appraisal checklist for quasi‐experimental studies (Barker et al. 2024). All studies were appraised by two reviewers (TH and YL), with any disagreements resolved by a third reviewer (MH).

## 3 Results

Database searching yielded 2497 records. After removing duplicates (*n* = 843), the titles and abstracts of 1654 records were reviewed, and 1611 records were excluded, leaving 43 records for full‐text review. Ultimately, eight papers were eligible and retained for this review.

### 3.1 Study Characteristics

The eight studies were published between 2002 and 2023 and were conducted in the United States (*n* = 4) (Gustafson et al. 2019; Nguyen et al. 2024; Van Orden et al. 2023; Zaslavsky et al. 2022), the Netherlands (*n* = 2) (Christie et al. 2022; Droes et al. 2004), the United Kingdom (*n* = 1) (Charlesworth et al. 2008), and a muti‐center study including Italy, Poland, and UK (*n* = 1) (Evans et al. 2020) (Table 1). Among these, three were definitive RCTs, and five were quasi‐experimental. In five studies, the control group received usual care or standard services (Charlesworth et al. 2008; Droes et al. 2004; Eloniemi‐Sulkava et al. 2002; Evans et al. 2020; Nguyen et al. 2024), while one study utilized an active control (limited intervention consisting of a book) (Gustafson et al. 2019). Sample sizes ranged from 12 to 316. All included interventions were psychosocial.

**TABLE 1:** Characteristics of reduction of loneliness interventions in informal caregivers of people with dementia (*N* = 8).

No	Author, year	Location	Age/Mean (Sd)	Study design	Sample size	Duration	Follow‐up	Intervention group	Control group	Measurement	Dementia type
1	Zaslavsky et al. 2022	US	65.8 (8.3)	Single‐arm, pre‐post pilot study	12	8 weeks	Weeks 9 and 10	Social networking intervention	No control group	UCLA loneliness scale	Lewy body dementias
2	Van Orden et al. 2023	US	61.37 (8.51)	Single‐arm, pre‐post pilot study	30	Less than 3 months	3 months	Psychotherapy	No control group	UCLA loneliness scale version 3	Alzheimer's disease and related dementias
3	Evans et al. 2020	Italy, Poland and UK	Intervention group 64.2 (13.3) Control Group: 64.2 (13.9)	Quasi experimental study	167	6 Months	—	Meeting centre support	Usual care	The 3‐item UCLA loneliness scale	Mild to moderate dementia
4	Gustafson et al. 2019	US	Intervention group: 69.7 (5.5) control Group: 68.6 (5.02)	RCT	31	6 Months	—	D‐CHESS intervention	Limited intervention consisting of a book	UCLA loneliness scale	Alzheimer's disease
5	Nguyen et al. 2024	US	68 (12)	Quasi experimental study	124	7 Months	—	Remote intervention	Usual care	The 3‐item UCLA loneliness scale	Alzheimer's disease
6	Christie et al. 2022	Netherlands	Intervention group: 58.1 (11.8) control group: 55.7 (13.6)	RCT	96	4 Months	4 Months	Online social support		The 1‐item loneliness scale	Dementia of all subtypes and stages
7	Droes et al. 2004	Netherlands	Intervention group: 63.6 (13.3) Control group: 60.9 (12.7)	Quasi experimental study	55	18 months	3 months, 7 months	Informative meetings and discussion group	Regular day‐care	The loneliness scale	Mild‐to‐moderate dementia
8	Charlesworth et al. 2008	UK	68 (11.4)	RCT	316	6 months	6, 15 and 24 months	Provide companionship and conversation	Usual care	The two‐item measure of emotional loneliness	Alzheimer's disease

### 3.2 Risk of Bias in Studies

The risk of bias assessment was performed for three RCTs and five quasi‐experimental studies. Using RoB 2.0 (Table 2), one study was rated as “low risk of bias”, and two RCTs were rated as having “some concerns”. Poor quality was often due to limited or absent details on randomization, concealment of group allocation, lack of intention‐to‐treat analysis, or failure to report blinding of outcome assessors. Using the revised JBI critical appraisal checklist, results for quasi‐experimental studies are presented in Table 3. Only one study met all the appraisal criteria. For single‐group, pre‐test/post‐test studies, the timing of post‐intervention data collection ranged from immediately after the intervention to 7 months following completion.

**TABLE 2:** Risk of bias assessment of the included RCTs (*n* = 3).

Study	Risk of bias domain					Overall risk
	Domain 1: Randomization process	Domain 2: Deviations from the intended intervention	Domain 3: Missing outcome data	Domain 4: Measurement of the outcome	Domain 5: Selection of the reported result	
Gustafson et al. 2019	Low	Some concerns	Some concerns	Low	Low	Some concerns
Christie et al. 2022	Low	Some concerns	Low	Low	Low	Some concerns
Charlesworth et al. 2008	Low	Low	Low	Low	Low	Low

**TABLE 3:** Risk of bias assessment of the included quasi‐experimental studies (*N* = 5).

Study	Temporal precedence	Selection and allocation	Confounding factors	Administration of intervention/exposure	Assessment, detection, and measurement of the outcome			Participant retention	Statistical conclusion validity
	Q1	Q2	Q3	Q4	Q5	Q6	Q7	Q8	Q9
Zaslavsky et al. 2022	UC	N	Y	UC	Y	Y	UC	Y	Y
Van Orden et al. 2023	UC	N	Y	Y	Y	Y	Y	UC	Y
Evans et al. 2020	N	Y	Y	UC	Y	Y	Y	UC	Y
Nguyen et al. 2024	UC	N	Y	UC	Y	Y	UC	UC	Y
Droes et al. 2004	UC	Y	N	UC	Y	Y	Y	UC	Y

### 3.3 Intervention Characteristics

The intervention characteristics of the included studies, following the TIDier checklist, are depicted in Table 4.

**TABLE 4:** Characteristics of intervention in included studies according to the TIDieR checklist (*n* = 9).

No	Authors	Why	What materials	What procedures	Who provided	How	Where	When and how much	Tailoring	Modifications	How well
1	Zaslavsky et al. 2022	To help caregivers of people with Lewy body dementia (LBD) address challenges they have experienced, with the end goal of reducing psychological distress in this population	A web‐based platform	The first 3 weeks included 8 individual interviews and 2 focus groups. The next 5 weeks involved psychoeducational materials based on problem‐solving therapy to identify relevant topics for the intervention	Advanced practice nurses who received moderator training from the study team	Digital discussion platform	Remote delivery	Duration: 8 weeks Frequency: once a week	Training and moderated web‐based discussion components	The intervention was adapted from a prior intervention for older adults to meet the needs of caregivers of individuals with LBD	Promising feasibility and preliminary efficacy data. 20% drop rate
2	Van Orden et al. 2023	To reduce loneliness and increase social connection for older Alzheimer's disease and related dementias caregivers experiencing stress and loneliness	8 individual coaching sessions include action planning to increase social connection, addressing barriers to social engagement, and developing problem‐solving skills	Caregivers identified specific goals related to social connection, created action plans to achieve these goals, and addressed barriers to implementation	Engage Coaches were doctoral level clinical psychologists and graduate students in clinical psychology	Video calls or phone calls	Remote delivery	Duration: 8 weekly coaching sessions over no more than 3 months Frequency: once a week	Tailored to the individual needs of each caregiver, focusing on addressing their specific barriers to social connection and personalizing action plans	Adapted from Engage Psychotherapy to specifically address the needs and challenges faced by dementia caregivers experiencing loneliness	Feasible and acceptable
3	Evans et al. 2020	The study aimed to implement and validate the Dutch Meeting Centre Support Program (MCSP) for family caregivers of people with dementia in Italy, Poland, and the UK	Not reported	Expert guest speakers provide informative/educational meetings covering various topics. The Meeting Centre Manager leads monthly discussion groups along with an external expert	A manager with relevant health and social care qualifications and experience, and expert guest speakers	Psychosocial interventions are provided in a friendly manner	Community locations	Duration: 6 months Frequency: three or less days a week	Adapted to meet the cultural and contextual requirements in each country, allowing for variations in intervention delivery based on local needs	Some modifications were made to the MCSP model to fit the diverse needs of participants	Not reported
4	Gustafson et al. 2019	To improve the lives of caregivers	Commercially available sensors, including a Bluetooth tracker, a GPS location tracker, and a motion sensor	The intervention group received access to the dementia‐Comprehensive Health Enhancement Support System website	Researchers	Caregivers received in‐home training for the system and sensor setup by the research staff	Remote delivery	Duration: 6 months	Participants received specific services and information tailored to their needs as family caregivers of participants	Feedback from participants suggested potential changes for future development	Not reported
5	Nguyen et al. 2024	To examine the effects of technology on caregiver loneliness and well‐being, as well as their technology experiences, during the COVID‐19 pandemic	WIFI‐enabled Samsung Galaxy tablets that are pre‐programmed with iN2L proprietary software that is designed for older adults	Family caregivers received training on how to use the tablets, attended group support sessions virtually, and accessed Alzheimer's association programs both live and on‐demand	Florida Department of Elder Affairs and senior engagement technology provider and Alzheimer's association	Video calls through the tablets	Remote delivery	Duration: 7 months Frequency: once per month for 1 h	The tablet content can be tailored to the older adult's specific likes and interests	Monthly support groups, newsletters, and access to virtual educational programs to enhance caregiver engagement and support	Not reported
6	Christie et al. 2022	To enhance social support and positive interactions in informal support networks, ultimately improving caregiver well‐being	The Inlife platform	The intervention group had access to the Inlife platform, while the control group received care as usual	Not reported	Smartphones and tablets	Remote delivery	Duration: 4 months	The Inlife platform allowed caregivers to personalize their profiles and invite specific individuals to their support circles	No	Not reported
7	Droes et al. 2004	To test the effectiveness of Meeting Centres Support in reducing the feelings of burden of carers	Informative meetings, discussion groups, day‐care activities for persons with dementia, psychomotor therapy, personal advice sessions, and monthly center meetings	The carers and persons with dementia participated in the MCS Program, receiving a combination of informative support, emotional support, and social support tailored to their individual needs	Permanent staff of professionals in general socio‐cultural community centers	Face‐to‐face	Community meeting centers	Duration: 7 months	The intervention was tailored to the individual needs of each client‐carer dyad based on their specific situation and requirements	No	31% dropped out
8	Charlesworth et al. 2008	To determine whether a social support intervention is effective compared with usual care alone	Full information booklet about using the befriending scheme	Befriending volunteers provide companionship and conversation, to be a listening ear and provide emotional support to the carer	Trained lay volunteer	Face‐to‐face	Home	Duration: 6 months or more Frequency: weekly	Tailored to each carer by matching them with a trained lay befriender based on their needs and preferences	No	No

Why? Only two studies provided the rationale for designing the intervention (Droes et al. 2004; Van Orden et al. 2023); the others lacked an underlying theoretical basis.

What materials? The materials varied across studies. Four studies provided digital devices, including tablets, Bluetooth trackers, GPS trackers, and online social support platforms. Three studies offered educational materials or prompts (Charlesworth et al. 2008; Van Orden et al. 2023; Zaslavsky et al. 2022).

What Procedures? Procedures included digital interventions, coaching, problem‐solving activities, and support sessions. Methods ranged from web‐based discussions to personalized coaching and technology facilitating caregiver support.

Who provided? The interventions were delivered by diverse professionals, including nurses, psychologists, clinical psychologists, dementia family care coordinators, meeting center managers, guest speakers, volunteers, and technology providers.

How? Five studies used digital platforms, video calls, phone calls, and specialized software. This allowed caregivers to engage with the interventions from their homes or other convenient locations. Three studies offered informative meetings and discussion groups, companionship and conversation, or training courses (Charlesworth et al. 2008; Droes et al. 2004; Evans et al. 2020).

Where? Most of the interventions were delivered remotely, with three being conducted in community locations or at home (Charlesworth et al. 2008; Droes et al. 2004; Evans et al. 2020).

When and How Much? Interventions lasted from 2 to 18 months, with frequencies ranging from once a week to three times a week.

Tailoring. All the studies reported that interventions were tailored to the specific needs and preferences of caregivers by small teams of trained staff and volunteers in person‐centered care.

Modifications. Five studies reported modifications based on feedback and outcomes of the interventions. Adaptations catered to the unique needs of caregivers of individuals with dementia, with feedback from participants informing potential changes for future iterations. Three studies did not report modifications (Charlesworth et al. 2008; Christie et al. 2022; Droes et al. 2004).

How well? Two studies reported data on the feasibility, acceptability, and preliminary efficacy, noting improvements in depression, burden, stress, loneliness, and caregiving competence (Van Orden et al. 2023; Zaslavsky et al. 2022). Two studies reported dropout rates, while others did not report effectiveness or feedback (Droes et al. 2004; Zaslavsky et al. 2022).

### 3.4 Outcomes

#### 3.4.1 Loneliness Outcomes

All included studies assessed loneliness, although it was not always designated as the primary outcome. The outcomes of the included studies were assessed in terms of their effectiveness in reducing loneliness among caregivers. Seven studies reported no significant improvements in loneliness, except for one study (Van Orden et al. 2023). Van Orden et al.'s (Van Orden et al. 2023) study found that engaged coaching was a promising behavioural intervention to reduce loneliness measured by the UCLA Loneliness Scale Version 3 (standardized response mean [SRM] = 0.63) and enhance social connection. Zaslavsky et al.'s (Zaslavsky et al. 2022) study showed that participants experienced a mean decrease in loneliness scores from baseline to post‐intervention, measured by the short 3‐item version of the Revised UCLA Loneliness Scale. Though the study was not powered for significance, on average, participants still reported a decrease in loneliness.

#### 3.4.2 Other Outcomes

Other outcomes were not consistent across studies, including stress, depressive symptoms, anxiety, and caregiver burden.

Stress. Four studies demonstrated that the intervention had a positive effect on caregiver stress levels (Gustafson et al. 2019; Nguyen et al. 2024; Van Orden et al. 2023; Zaslavsky et al. 2022). Two studies reported that no significant improvements were demonstrated for stress (Christie et al. 2022; Droes et al. 2004).

Depressive symptoms. Only one pilot trial reported the positive effect of the intervention on depressive symptoms measured by the Center for Epidemiologic Studies Depression (CES‐D) Scale (Zaslavsky et al. 2022). One RCT demonstrated that only a small number of caregivers who participated in the befriender intervention reported a reduction in depression scores that approached statistical significance (95% CI: 0.09–2.84) (Charlesworth et al. 2008). Three studies did not show significant improvements in depressive symptoms (Christie et al. 2022; Gustafson et al. 2019; Van Orden et al. 2023).

Anxiety. One study reported potential improvements in anxiety measured by the Generalized Anxiety Disorder scale (Gustafson et al. 2019). However, three studies didn't show significant improvements in anxiety (Charlesworth et al. 2008; Christie et al. 2022; Van Orden et al. 2023).

Caregiver burden. One pilot study reported that participants exhibited an average improvement in burden related to caregiving responsibilities by using the full 22‐item version of the Zarit Caregiver Burden (Zaslavsky et al. 2022). One pre‐test and post‐test controlled trial showed that most family caregivers reported feeling less burdened and more supported after participating in the meeting center support program (Evans et al. 2020). Three studies reported the native effect on caregiver burden (Charlesworth et al. 2008; Droes et al. 2004; Gustafson et al. 2019).

## 4 Discussion

This is the first systematic review we know of to summarize the existing literature and describe the interventions aimed at reducing loneliness among informal caregivers of people with dementia. Although most studies did not present statistically significant results, this systematic review identified some promising areas for future studies. Remote interventions have shown promise by lowering participation barriers and increasing the acceptability of the interventions. These interventions also have potential benefits for improving caregivers' mental health and well‐being, such as depression, anxiety, stress, and caregiver burden.

Remote intervention was the most frequently utilized method, and the results indicate that it may improve caregivers' ability to care for people with dementia and help them better understand the disease, especially given the heightened salience of social isolation during the COVID‐19 pandemic (Nguyen et al. 2024; Zaslavsky et al. 2022). Integrating remote interventions in caregiving support holds immense promise in overcoming geographical barriers and enhancing accessibility for caregivers, particularly those in remote or underserved areas. In addition to enhancing access and support, remote interventions have improved caregiver satisfaction and engagement (Van Orden et al. 2023). This aligns with the findings of a systematic review, which indicates that remote interventions for family caregivers have become increasingly effective in recent years, addressing the unique needs of caregivers and their care recipients (Graven et al. 2021).

In our review, the interventions identified were psychosocial approaches, and they have the potential to significantly enhance caregivers' mental health and well‐being, such as depression, anxiety, and stress. Research indicates that informal caregivers' loneliness can negatively impact their mental health and caregiving abilities (Saadi et al. 2021). This loneliness is closely associated with mental health outcomes, as caregivers often report heightened levels of depression and anxiety due to their isolation (Victor et al. 2021). The emotional burden of caregiving was especially severe among those caring for individuals with moderate to severe dementia, who already experienced significant stress and emotional strain before the COVID‐19 pandemic (Azevedo et al. 2021). Programs designed to enhance social support and provide coping strategies have proven effective in reducing loneliness and improving mental health among caregivers (Livingston et al. 2013; Zaslavsky et al. 2022). These interventions not only aim to alleviate loneliness but also strengthen caregivers' resilience and coping mechanisms, which are crucial for maintaining their well‐being and the quality of care they deliver (Harmell et al. 2011). Additionally, fostering meaningful relationships and community connections can mitigate the negative psychological effects of caregiving, thereby promoting better mental health outcomes (Eloniemi‐Sulkava et al. 2002; Victor et al. 2021).

Several factors may explain the ineffectiveness of some interventions. First, many interventions lack a theoretical basis, which limits their effectiveness. In this review, only two studies provided a clear rationale for their interventions (Droes et al. 2004; Van Orden et al. 2023). Understanding the mechanisms through which interventions reduce loneliness is crucial for developing effective strategies. For example, social support interventions may work by enhancing social skills, providing opportunities for social interaction, and addressing maladaptive social cognitions (Zagic et al. 2022). However, the effectiveness of these mechanisms can vary depending on individual caregiver needs and circumstances. Second, more intensive or longer‐term interventions may be necessary to produce meaningful change (Zaslavsky et al. 2022). Third, social determinants of health, such as socioeconomic status or access to technology, may also influence the effectiveness of interventions. Last, the requirement for internet access and technological proficiency excludes many potential participants, particularly those from lower socioeconomic backgrounds (Grošelj et al. 2019).

The review identified several limitations present in the included studies. The study design and power were compromised by the lack of control groups and the absence of randomization, which limited the robustness and statistical significance of the findings (Zaslavsky et al. 2022). The studies also suffered from a lack of racial, ethnic, and gender diversity among participants, limiting the generalizability of the results (Droes et al. 2004). Small sample sizes and restricted geographic distributions further constrained the applicability of the findings to broader populations (Eloniemi‐Sulkava et al. 2002; Zaslavsky et al. 2022). Many studies did not assess the long‐term sustainability of the observed benefits, and reliance on self‐reported data introduced potential biases, affecting the accuracy of the reported psychological states.

Future work should include larger RCTs to provide robust evidence for intervention effectiveness. Efforts to diversify samples in race, ethnicity, gender, and socioeconomic status, including participants from various regions, will enhance generalizability. Addressing technological barriers by providing support such as internet access and digital tool training is essential. Additionally, longitudinal studies with extended follow‐up periods are needed to assess the sustainability of intervention benefits. Practical implementation should be explored by delivering interventions through a broader range of professionals, including those in community agencies and primary care settings, to improve scalability. Future studies should also consider the cost‐effectiveness of interventions, as understanding the economic impact is crucial for implementation (Charlesworth et al. 2008). Finally, research should investigate cultural and contextual adaptations to better meet the needs of diverse caregiver populations, considering the influence of race, ethnicity, and cultural factors on experiences and outcomes.

## 5 Implications

The implications of this systematic review suggest that while remote interventions have practical appeal by providing accessibility and convenience, they must be carefully tailored to individual caregiver needs, including considerations for their technological proficiency and personal preferences. Additionally, future studies should prioritize tracking long‐term effects to ascertain sustained mental health benefits for caregivers. This approach could inform the development of guidelines and standards for intervention implementation, ensuring that caregivers receive consistent and reliable support. By focusing on these areas for improvement and leveraging the promise shown by remote interventions, stakeholders can better address the multifaceted issue of caregiver loneliness, ultimately enhancing the quality of life for both caregivers and the individuals with dementia they support.

For nursing and clinical practice, these findings highlight the importance of integrating personalized remote interventions into care plans for informal caregivers. Nurses and healthcare professionals can play a crucial role in assessing caregiver needs, providing appropriate training for technology use, and offering ongoing support to maximize the effectiveness of these interventions. Ultimately, this will enhance the quality of life for both caregivers and the individuals with dementia they support, fostering a more holistic and supportive care environment.

## 6 Strengths and Limitations

One strength of this systematic review is that it is the first study to examine the efficacy of interventions for loneliness among informal caregivers of dementia patients and describe the characteristics of these interventions. It has highlighted the need for an appropriate rationale that helps to define interventions based on the mechanisms. Another strength is the comprehensive search and standardized data extraction and screening procedures, in‐depth descriptions, and data synthesis.

We also acknowledge that our review has some limitations. First, we included only English studies, which may have resulted in the omission of other potentially relevant research. Second, a meta‐analysis was not conducted due to the small sample sizes and varied study designs, as well as the fact that most of the study results were not statistically significant.

## 7 Conclusion

This systematic review underscores the potential benefits of interventions aimed at reducing loneliness among informal caregivers of dementia patients, although most studies did not achieve statistically significant results. Remote interventions showed promise by reducing participation barriers and enhancing acceptability. Individualized and structured multicomponent interventions emerged as promising approaches to mitigate caregiver burden. However, the studies faced several limitations, including compromised study designs, lack of diversity, small sample sizes, and technological barriers. Future research should focus on larger randomized controlled trials to establish robust evidence, diversify participant demographics, address technological challenges, and explore the long‐term sustainability of intervention benefits.

## Author Contributions

Tianxue Hou: conceptualization, investigation, methodology, data curation, writing – original draft. Yuqian Luo, Mu‐Hsing Ho, Xiaohang Liu, and Yuelin Li: validation, writing – review and editing. Chia‐Chin Lin: supervision, validation, writing – review and editing.

## Funding

The authors have nothing to report.

## Ethics Statement

The review protocol is registered with PROSPERO (CRD42024500648). Further details can be accessed at the PROSPERO website (https://www.crd.york.ac.uk/prospero/display_record.php?RecordID=500648).

## Conflicts of Interest

The authors declare no conflicts of interest.

## Supporting information


**Data S1:** PRISMA_2020_checklist.

**Data S2:** Search strategy.

## Data Availability

Data sharing not applicable to this article as no datasets were generated or analysed during the current study.
