# Supplementary material for: Interventions to Reduce Loneliness in Informal Caregivers of People With Dementia: A Systematic Review
Source: J Clin Nurs. 2026 Jun 2;35(10):4124–36. doi: 10.1111/jocn.70382 (PMC13569374; doi:10.1111/jocn.70382)
Supplement: Supplementary file 2 [file JOCN-35-4124-s002.docx]

**Search Strategy**

| **1. MEDLINE via PubMed** | |
| --- | --- |
| #1 | cognition [MeSH Terms] OR dementia [MeSH Terms] OR “cognition disorders” [Title/Abstract] OR “Alzheimer*” [Title/Abstract] OR “dement*” [Title/Abstract] OR “cogni*” [Title/Abstract] |
| #2 | Spouses [MeSH Terms] OR “Adult Children” [MeSH Terms] OR Family [MeSH Terms] OR “caregiver*” [Title/Abstract] OR “caregiv*” [Title/Abstract] OR “carer*” [Title/Abstract] OR caring [Title/Abstract] OR “famil*” [Title/Abstract] OR “caretaker*” [Title/Abstract] OR “spouse*” [Title/Abstract] OR home [Title/Abstract] OR “relative*” [Title/Abstract] |
| #3 | Loneliness [MeSH Terms] OR alone* [Title/Abstract] OR lonel* [Title/Abstract] OR living alone [Title/Abstract] |
| #4 | treatment [Title/Abstract] OR experiment* [Title/Abstract] OR intervention [Title/Abstract] OR therapy [Title/Abstract] OR program [Title/Abstract] |
